# Microwave-Assisted Synthesis of Novel 2,3-Dihydro-4-Pyridinones

**DOI:** 10.3390/molecules15118425

**Published:** 2010-11-17

**Authors:** Bahjat A. Saeed, Rita S. Elias, Wisam A. Radhi

**Affiliations:** 1 Department of Chemistry, College of Education, University of Basrah, Iraq; 2 Department of Pharmaceutical Chemistry, College of Pharmacy, University of Basrah, Iraq

**Keywords:** curcumin, microwave-assisted synthesis, Montmorillonite K-10, dihydropyridones

## Abstract

Novel 2,3-dihydro-4-pyridinones were synthesized via the reaction of curcumin and primary amines or amine acetates under microwave irradiation. Montmorillonite K-10 was used as a catalyst. Reaction times did not exceed 120 s. The structures of the compounds were established by elemental analysis and from their mass, ^1^H- and ^13^C-NMR spectra.

## 1. Introduction

Dihydropyridones are important intermediates for the synthesis of natural products, particularly alkaloids, and have been investigated extensively as valuable building blocks for the construction of piperidines, perhydroquinolines, indolizidines, quinolizidines and other alkaloid ring systems possessing a wide range of biological and pharmacological properties [1,2,3,4,5]. For their synthesis, the addition of Grignard reagents to 1-acyl-4-methoxy pyridinium salts has been exploited by Commins [6,7,8,9,10]. Hetero- Diels-Alder reactions or stepwise, formal [4+2] transformations involving imines have also been employed [11,12,13]. Recently they have been synthesized via cyclization of α,β-unsaturated 1,3-diketones in acidic medium [14] and through catalytic metathesis of *o*-alkynylanilines and aldehydes [15]. A facile route to functionalized dihydropyridones has been developed via formal [5C+1N] annulations of α-alkynoyl ketene-(*S*,*S*)-acetals with aliphatic amines [16]. In addition, partial reduction of pyridinium salts has also been exploited for their synthesis [17]. We previously reported the microwave-assisted formation of 2,3-dihydro-4-pyridinones from curcumin and simple primary amines in the presence of Montmorillonite K-10 via a transient imine [18,19]. Curcumin is an α,β-unsaturated 1,3-diketone that constitutes one of the three major components of the Indian herb *Curcuma longa* [20,21]. In continuation of our interest in the reactions of unsaturated 1,3-diketones and amines for the synthesis of dihydropyridones under microwave irradiation, we report herein the microwave-assisted synthesis of novel dihydropyridones from the reaction of curcumin and primary amines or amine acetates. The reported compounds had not previously been obtained by conventional methods.

## 2. Results and Discussion

**Scheme 1:**
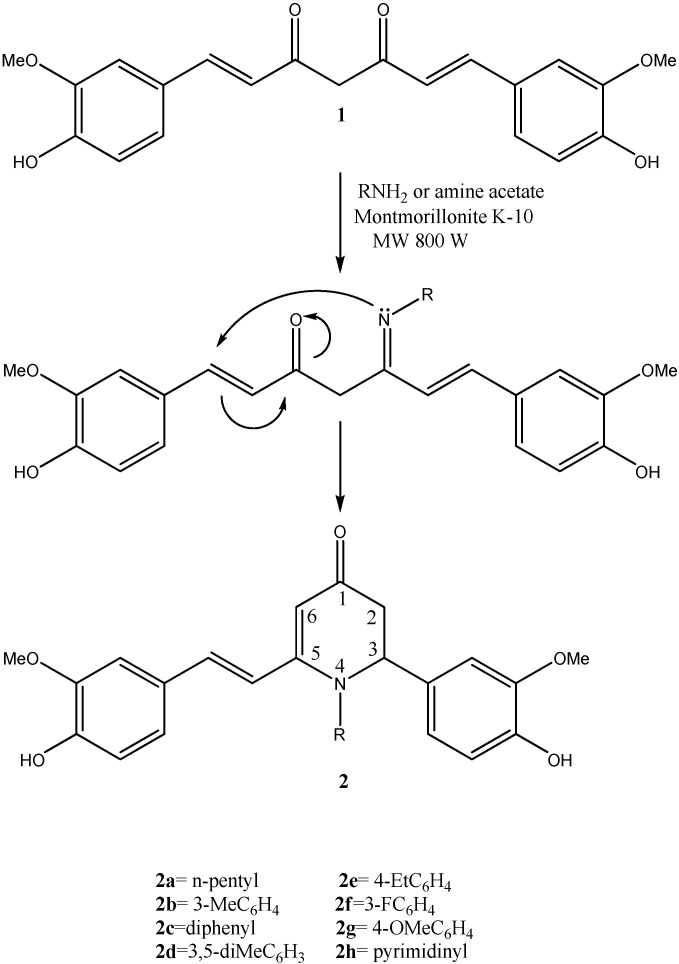
The mechanism, reaction conditions and prepared compounds.

The dihydropyridones were synthesized by microwave-assisted reaction of curcumin with either primary amines (*n*-pentylamine) or amine acetates (all aromatic amines) in the presence of Montmorillonite K-10 as the catalyst. The experimental procedure involved absorbing the reactants on Montmorillonite K-10, then irradiating the mixture with microwaves. The product was extracted from the clay with ethanol and the compounds were separated by column and then by preparative TLC chromatography. The reaction time was 120 s and the yields ranged from 17 to 28%. Attempts to enhance the yields by using longer reaction times proved unsuccessful. The dihydropyridones were synthesized via transient imine mechanism (Scheme 1) and were confirmed by an elemental analysis and by mass, ^1^H- and ^13^C-NMR spectroscopy.

The ^1^H-NMR spectra of the products showed two singlets within the 8.91-9.04 and 9.38-99.44 ppm ranges assigned to the two OH groups occupying different chemical environments, which proved the asymmetrical structure of the products. In contrast the ^1^H-NMR spectrum of symmetrical curcumin contained only one singlet at 9.79 ppm for the OH group proton. The spectra are also characterized by a one proton singlet in the 5.01-5.45 ppm range assigned to the H6 vinylic proton. In addition, two doublets of doublets were also apparent within the 2.40-2.65 ppm (*J* ≈ 16 and 4 Hz) and 2.80-3.07 ppm (*J* ≈ 16 and 7 Hz) range which were assigned to two geminal protons (C-2) coupled to each other, as was confirmed by the HOMO-COSY spectra. Further, the HETCOR spectra indicated that these protons were attached to the same carbon atom (C-2) and each was coupled to the methine proton (3-H), which occurred as a multiplet within the 4.66-5.12 ppm range. The protons of the methylene group attached to the nitrogen in the *N*-pentyl substituted compound were diastereotopic, and appeared as two multiplets at 3.05 and 3.74 ppm. The HETCOR spectrum of this compound showed that these signals belong to protons connected to the same carbon (C2) which has a signal at 51.5 ppm. ^13^C-NMR spectra revealed the C=O signals within the 190.0-187.9 ppm range. UV-vis spectra (in ethanol) of the products were characterized by a band within the 338-366 nm range, which was strongly blue-shifted (ca. 60 nm) compared to curcumin whose band appears at 420 nm. This blue shift reflects the reduction of conjugation in the products due to participation of one of the olefinic groups of curcumin in the ring-closure to give the corresponding dihydropyridone.

## 3. Experimental

### 3.1. General

NMR spectra were recorded on a Bruker 400 MHz spectrometer in deuterated DMSO with tetramethylsilane as an internal standard. Mass spectra were determined on a Shimadzu GCMS-QP 1000 EX instrument at 70 eV. Melting points were determined in open capillary tubes in a Buchi-510 apparatus. Elemental analyses were performed by Thermo Finnigan CHNS-O analyzer, 1112 series.

### 3.2. General Method for the Synthesis of Dihydropyridones

The method described by Elias *et al.* [18] was employed. Curcumin (2 g, 5.4 mmol) and Montmorillonite K-10 (3 g) were mixed in a mortar and placed in a 10 mL beaker. The appropriate amount of amine or amine acetate (5.4 mmol) was added to the mixture, which was then thoroughly mixed. The mixture was irradiated in a commercial microwave oven (Samsung 800 MW) for 120 s at 800 W. The reaction protocol includes the irradiation of the reactants for 10 s then cooling to room temperature and mixing. This was repeated until 120 s of reaction time were accumulated. The extent of reaction was monitored by TLC using THF/chloroform (30:70) as eluent. On completion, the mixture was extracted with EtOH (5 × 3 mL). The Montmorillonite was removed by filtration and the solvent was evaporated. The products were separated by column chromatography (silica gel) using THF/chloroform (1:5) as eluent. The product fractions were further separated by preparative TLC (silica gel) using the same eluent. The dihydropyridones were obtained as yellow powders. 

*2-(4-hydroxy-3-methoxyphenyl)-6-(4-hydroxy-3-methoxystyryl)-1-pentyl-2,3-dihydropyridin-4(1H)-one* (**2a**). Yield 28%; m.p. 244-246 °C; EI-MS: *m/z* = 437 (M^+^); ^1^H-NMR δ 0.59 (t, *J* = 6.0 Hz, 3H,N-(CH_2_)_4_-CH_3_), 1.18 (m, 4H, N-CH_2_-(CH_2_)_2_), 1.49 (m, 2H,N-CH_2_-CH_2_), 2.42 (dd, *J* = 16.0 and 4.0 Hz, 1H, 2-H), 2.84 (dd, *J* = 16.0 and 7.0 Hz, 1H, 2-H), 3.05 (m, 1H, NCH_2_),3.74 (s, 4H, NCH_2_ + OCH_3_), 3.82 (s, 3H, OCH_3_), 4.66 (m, 1H,3-H), 5.09 (s, 1H, 6-H), 6.65-7.29 (m, 8H, olefinic + Ar-H), 8.97 (s, 1H, OH), 9.37 (s, 1H, OH); ^13^C-NMR δ 13.8, 21.8, 27.3, 28.7, 41.9, 51.5, 55.6, 55.7, 60.5, 95.8, 111.9, 112.3, 115.2, 115.4, 115.5, 119.0, 119.7, 121.7, 123.0, 127.2, 130.2, 131.6, 134.5, 135.3, 137.1, 145.0, 145.8, 146.7, 160.9, 187.9; Anal. Calcd. for C_26_H_31_NO_5_: C, 71.37; H, 7.14; N, 3.20. Found: C, 71.43; H, 7.30; N, 3.16.

*2-(4-hydroxy-3-methoxyphenyl)-6-(4-hydroxy-3-methoxystyryl)-1-m-tolyl-2,3-dihydropyridin-4(1H)-one* (**2b**). Yield 19%; m.p. 190 °C; EI-MS: *m/z* = 457 (M^+^); ^1^H-NMR δ 2.22 (s, 3H, Ar-CH3), 2.62 (dd, *J* = 16 and 4 Hz, 1H, 2-H), 3.07 (dd, *J* = 16.0 and 6.0 Hz, 1H, 2-H), 3.70 (s, 3H, OCH_3_), 3.71 (s, 3H, OCH_3_), 5.10 (m, 1H, 3-H), 5.43 (s, 1H, 6-H), 6.29-7.22 (m, 12H, olefinic + Ar-H), 8.96 (s, 1H, OH), 9.41 (s, 1H, OH); ^13^C-NMR δ 12.7, 17.6, 19.3, 31.2, 42.8, 49.7, 60.3, 95.8, 115.3, 116.0, 118.4, 127.9, 129.2, 129.3, 137.1, 153.4, 157.3, 159.3, 162.2, 189.2; Anal. Calcd. for C_28_H_27_NO_5_: C, 73.51; H, 5.95; N, 3.06. Found: C, 73.36; H, 6.21; N, 2.86.

*1-(biphenyl-4-yl)-2-(4-hydroxy-3-methoxyphenyl)-6-(4-hydroxy-3-methoxystyryl)-2,3-dihydropyridin-4(1H)-one* (**2c**). Yield 16%; m.p. 190 °C; EI-MS: *m/z* = 519 (M^+^); ^1^H-NMR δ 2.61 (dd, *J* = 16.4 and 4.4 Hz, 1H, 2-H), 3.04 (dd, *J* = 16.4 and 6.0 Hz, 1H, 2-H), 3.71 (s, 6H, OCH_3_), 5.03 (m, 1H, 3-H), 5.40 (s, 1H, 6-H), 6.30 (d, *J* = 16 Hz, 1H, olefinic-H), 6.85-7.22 (m, 15H, olefinic + Ar-H), 8.98 (s, 1H, OH), 9.46 (s, 1H, OH); ^13^C-NMR δ 43.1, 55.5, 55.6, 64.3, 98.4, 111.1, 115.2, 115.7, 116.8, 119.1, 119.9, 120.5, 120.9, 126.8, 128.8, 129.1, 130.4, 136.0, 141.5, 143.1, 145.8, 147.7, 148.2, 185.4, 189.1; Anal. Calcd. for C_33_H_29_NO_5_: C, 76.28; H, 5.63; N, 2.70. Found: C, 77.08; H, 5.80; N, 2.86.

*1-(3,5-dimethylphenyl)-2-(4-hydroxy-3-methoxyphenyl)-6-(4-hydroxy-3-methoxystyryl)-2,3-dihydropyridin-4(1H)-one* (**2d**). Yield 19%; m.p. 190 °C; EI-MS: *m/z* = 471 (M^+^); ^1^H-NMR δ 2.18 (s, 6H, Ar-CH_3_), 2.60 (dd, *J* = 16.4 and 3.2 Hz, 1H, 2-H), 3.07 (dd, *J* = 16.4 and 6.4 Hz, 1H, 2-H), 3.70 (s, 3H, OCH_3_), 3.71 (s, 3H, OCH_3_), 5.08 (m, 1H, 3-H), 5.42 (s, 1H, 6-H), 6.33 (d, *J* = 16.0 Hz, 1H, olefinic-H), 6.70-6.97 (m, 9H, Ar-H), 7.19 (d, *J* =16.0, 1H, olefinic-H), 8.98 (s, 1H, OH), 9.38 (s, 1H, OH); ^13^C-NMR δ 20.8, 42.7, 55.5, 55.6, 64.1, 100.2, 110.9, 111.2, 115.2, 115.7, 118.8, 120.8, 120.9, 122.7, 127.1, 130.1, 136.2, 138.1, 144.5, 145.7, 147.5, 147.7, 148.3, 157.4, 189.5; Anal. Calcd. for C_29_H_29_NO_5_: C, 73.87; H, 6.20; N, 2.97. Found: C, 74.11; H, 6.04; N, 3.19.

*1-(4-ethylphenyl)-2-(4-hydroxy-3-methoxyphenyl)-6-(4-hydroxy-3-methoxystyryl)-2,3-dihydropyridin-4(1H)-one* (**2e**). Yield 15%; m.p. 190 °C; EI-MS: *m/z* = 471 (M^+^); ^1^H-NMR δ 1.13 (t, *J* = 6.0 Hz, 3H, Ar-CH_2_-CH_3_), 2.49 (q, *J* = 6.0 Hz, 2H, Ar-CH_2_), 2.54 (dd, *J* = 16.0 and 4.1 Hz, 1H, 2-H), 3.07 (dd, *J* = 16 and 6 Hz, 1H, 2-H), 3.71 (s, 3H, OCH3), 3.72 (s, 3H, OCH_3_), 5.07 (m, 1H, 3-H), 5.40 (s, 1H, 6-H), 6.23 (d, *J* = 15.6 Hz, 1H, olefinic-H), 6.71-7.18 (m, 11H, olefinic + Ar-H), 9.0 (broad, 2H, OH); ^13^C- NMR δ 15.4, 27.5, 40.1, 42.9, 55.5, 55.6, 64.2, 99.6, 111.0, 111.1, 115.2, 115.8, 118.8, 120.0, 121.4, 125.1, 127.3, 128.3, 130.2, 136.5, 141.2, 142.2, 145.8, 147.6, 148.0, 157.9, 189.3; Anal. Calcd. for C_29_H_29_NO_5_: C, 73.87; H, 6.20; N, 2.97. Found: C, 73.66; H, 6.32; N, 2.76.

*1-(3-fluorophenyl)-2-(4-hydroxy-3-methoxyphenyl)-6-(4-hydroxy-3-methoxystyryl)-2,3-dihydropyridin-4(1H)-one* (**2f**). Yield 19%; m.p. 190 °C; EI-MS: *m/z* = 461 (M^+^); ^1^H-NMR δ 2.68 (dd, *J* = 16.6 and 4.0 Hz, 1H, 2-H), 3.07 (dd, *J* = 16.6 and 6.0 Hz, 1H, 2-H), 3.71 (s, 3H, OCH_3_), 3.72 (s, 3H, OCH_3_), 5.2 (m, 1H, 3-H), 5.47 (s, 1H, 6-H), 6.30 (d, *J* = 16.1 Hz, 1H, olefinic-H),6.60-7.38 (m, 11H, olefinic + Ar-H), 8.95 (s, 1H, OH), 9.36 (s, 1H, OH); ^13^C-NMR δ 42.8, 55.6, 64.2, 101.9, 110.9, 111.3, 121.1, 115.7, 118.8, 119.7, 120.5, 121.1, 127.0, 129.8, 130.4, 136.8, 145.8, 146.2, 147.6, 147.8, 148.3, 157.1, 160.8, 163.2, 190; Anal. Calcd. for C_28_H_27_NO_5_: C, 70.27; H, 5.24; N, 3.04. Found: C, 70.36; H, 5.40; N, 2.82.

*2-(4-hydroxy-3-methoxyphenyl)-6-(4-hydroxy-3-methoxystyryl)-1-(4-methoxyphenyl)-2,3-dihydropyridin-4(1H)-one* (**2g**). Yield 17%; m.p. 190 °C; EI-MS: *m/z* = 473 (M^+^). ^1^H-NMR δ 2.65 (dd, *J* = 16.0 and 4.0 Hz, 1H, 2-H), 3.08 (dd, *J* = 16.0 and 6.0 Hz, 1H, 2-H), 3.71 (s, 6H, 2 × OCH_3_), 3.72 (s, 3H, OCH_3_), 5.07 (m, 1H, 3-H), 5.42 (s, 1H, 6-H), 6.29 (d, *J* = 16.0 Hz, 1H, olefinic-H), 6.60-7.22 (m, 11H, olefinic + Ar-H), 8.93 (s, 1H, OH), 9.40 (s, 1H, OH); ^13^C-NMR δ 40.0, 42.7, 55.6, 55.7, 64.8, 99.7, 111.0, 111.4, 115.3, 115.8, 119.0, 120.0, 121.2, 125.5, 127.5, 128.2, 130.4, 136.5, 141.2, 142.6, 146.0, 147.7, 148.3, 157.9, 189.5; Anal. Calcd. for C_28_H_27_NO_5_: C, 71.02; H, 5.75; N, 2.96. Found: C, 72.21; H, 5.60; N, 2.67.

*2-(4-hydroxy-3-methoxyphenyl)-6-(4-hydroxy-3-methoxystyryl)-1-(pyrimidin-4-yl)-2,3-dihydropyridin-4(1H)-one* (**2h**). Yield 18%; m.p. 190 °C; EI-MS: *m/z* = 445 (M^+^); ^1^H-NMR δ 2.65 (dd, *J* = 16.2 and 4.1 Hz, 1H, 2-H), 3.08 (dd, *J* = 16.2 and 6.0 Hz, 1H, 2-H), 3.71 (s, 3H, OCH_3_), 3.72 (s, 3H, OCH_3_), 5.06 (m, 1H, 3-H), 5.46 (s, 1H, 6-H), 6.29 (d, *J* = 16.0 Hz, 1H, olefinic-H),6.67-7.47 (m, 11H, olefinic + Ar-H), 9.06 (s, 1H, OH), 9.46 (s, 1H, OH); ^13^C-NMR 42.9, 55.6, 55.7, 64.6, 101.8, 110.9, 111.4, 121.3, 116.0, 118.8, 119.4, 120.7, 121.8, 125.6, 127.1, 130.6, 136.7, 146.4, 147.7, 148.1, 148.4, 158.1, 161.0, 163.7, 164.4, 190.0; Anal. Calcd. for C_25_H_273_NO_5_: C, 67.02; H, 5.20; N, 9.43. Found: C, 67.64; H, 5.29; N, 9.63.

## 4. Conclusions

In conclusion, this work has demonstrated the positive role of microwave irradiation in the synthesis of dihydropyridones from the reaction of curcumin and simple amines which could not be performed under traditional heating conditions. 
